# Notes and Comments

**Published:** 1924-04

**Authors:** 


### NOTES AND COMMENTS.

### WHAT CAUSES RHEUMATISM?

EVERYONE who is interested in preventive medicine, which ought to mean every intelligent person, will be grateful for the valuable report on "The Incidence of Rheumatic Diseases," which has just been published by the Ministry of Health. It is not only an admirable example of team-work, but an instructive example of what can be accomplished by that great body of general practitioners, the panel doctors—men who, we are sometimes tsked to believe, have little interest in preventive work, and little time in which to study the cumulative bearing of the cases that come before them. The report refers solely to insured persons, and is the first general and organised effort of the panel doctors to undertake a group inquiry. Their task was not easy. "Rheumatism" is a very inclusive word, and careful definitions were necessary. The result of the inquiry, although it has not fathomed the mystery of rheumatism, has been highly informative. It shows, for instance, that in the toll it lays upon industry, rheumatism is worse than strikes. It causes one-sixth of our industrial invalidity, loses three million weeks of work in a year and costs the Approved Societies nearly two millions of money in sick benefit. Chronic joint diseases alone account for one half of these figures. Women are more prone to rheumatic fever and rheumatoid arthritis than men; on the other hand sciatica, lumbago, and gout are infinitely more common among men than among women. There are, indeed, fifty gouty men to one gouty woman. We still do not know the first cause of any form of rheumatism, but we do know from this report that tonsillitis plays an important part in rheumatic fevers, and that dental sepsis is frequently associated with rheumatism of the joints. One encouraging detail of the report is that rheumatic fever is becoming less virulent as well as less common, the hospitals receiving only one half of the former number of cases. It is the rheumatism of every day life that is now the enemy. When we learn, as some day we shall learn, how to prevent it, there will be not only a vast saving of money, but an incalculable addition to the sum of human happiness.

### THE TRAFFIC IN NARCOTICS.

THERE is solid ground for hope that we are on the eve of a really effective agreement for the limitation of the manufacture of morphia, cocaine, and their salts. A special committee of experts under the League of Nations has just been sitting at Geneva to prepare the programme of the International Conference which next November is to consider the best means of squaring the production of these harmful drugs with the medical and scientific needs of the world. The efforts that have hitherto been made in the direction of limitation have not been successful. Hopes of reform have been based upon the Opium Convention of 1912 under which each State undertakes to limit to medical and other legitimate requirements the manufacture of drugs of addiction. Since, however, there has been no machinery for estimating the extent of these requirements the task of the Opium Committee, the official adviser of the League of Nations on the subject, has been so difficult as to be almost hopeless. Production is still much too great, so that a large surplus of these drugs is available for clandestine disposal. Now that the situation is much clearer than when the Opium Committee began work three years ago, we may reasonably expect that the Conference of next November will be able so to regulate the world's production and manufacture that the perils to health and life that are produced by the comparative ease with which narcotic drugs can at present be obtained will be so reduced as to become negligible.

### TOWN AND SUBURBAN HOSPITALS.

ONCE more the question has been raised of the wisdom of rebuilding hospitals in the centre of London and, presumably, of other great towns. It is pointed out that half a dozen of the great London Hospitals are situated within a small radius, that the bulk of the population lives in districts far removed from that radius, and that the great King's College Hospital has already betaken itself to Denmark Hill. That the sites of such historic institutions as St. Bartholomew's and Guy's could be sold for great sums of money is undoubted; but the matter is by no means so simple as it looks. To denude central London of its hospitals would be almost as unwise as to build fresh ones in the heart of the town, and a good deal more has to be considered than the opportunity of putting hospital finance on a sound footing at one stroke, important as that would be. For the patient, especially the out-patient, all roads lead to the centre, but all roads do not lead to a given suburb. And what applies to the patient applies very forcibly also to the visiting medical staffs. To the busy surgeon or consultant a journey, even by car, to a comparatively distant suburb makes a serious draft upon time. It is by no means improbable, however, that one or two of the great London hospitals will, sooner or later, remove from the centre to the circumference, and we may be sure that the future tendency of such institutions will everywhere be outwards. Business centres are ever growing wider, which means a reduction in the number of residential buildings and a swelling of the suburban population.

### A PARTISAN RENT BILL.

MR. B. GARDNER'S Bill to amend the Rent and Mortgage Interest Restrictions Act of last year is a frankly partisan measure, as its own prefatory memorandum rather cynically admits. Its object, the memorandum says, is to amend the existing Acts "in the interests of the tenants." It is not difficult to imagine the pious horror and the comminatory language of Mr. Gardner and his extremest friends if some one were to bring in a Bill admittedly conceived "in the interests of the landlords." It is not along partisan lines that we can hope to settle the twin questions of housing and controlled rents.

The provisions of the Bill, which has received a Second Reading in the House of Commons and is now before a Standing Committee, are simple and sweeping. It continues the Act of 1920 to 1928, greatly curtails the owner's power of regaining possession, reduces to nearly one half the increases that can be made in terms of combined rent and rates, and brings back into control all the houses which, by the operation of last year's Act, have been freed from restrictions. Thus to upset the working of a measure which was passed less than three-quarters of a year ago is in itself a wanton proceeding; nothing whatever is to be gained by the constant chopping and changing of the law. It is, unfortunately, only too probable that the Act which expires in the summer of next year will have to be extended in its duration, but we are not yet in a position to estimate what may be a reasonable extension. Control, evil as are its results, may have to be continued until a very much larger number of houses are available; but a measure of this type, if it became law, would inevitably seriously reduce the flow of bricks and mortar to the land. It would thus increase the scarcity and compel the almost indefinite prolongation of restrictions. Happily there is small likelihood of its reaching the statute-book in anything approximating to its original form. With the exception of the "wild men" from Glasgow its supporters are lukewarm, and although the Government appeared at first to incline towards it, their present attitude is that of a rather lukewarm "standing by."

### DECADENT MAN.

DR. JANE WALKER has been giving the modern Englishman a piece of her mind, and when women do that to men they usually do it thoroughly. The Englishman, she declares, is losing all his hardihood. He has a hot bath instead of a cold one, he is a chain-smoker of cigarettes, he wears spats, woolly waistcoats and fur coats (these latter meet only for "Company promoters"), he stays too long in bed and—at all events if he is married—has a cup of tea before he leaves it. It is "bad enough to wear thick gloves," but some young men actually have their hands manicured. For them no fitting words can be found. Our fondness for central heating is another sign of softness, while as for cushions —! Dr. Jane Walker never uses one. Were it not for the "ridiculous white plaster" of his evening shirt the current man would be entirely effeminate. Here we hardly follow Dr. Walker. The "white plaster" is so thick as to be an effective protector of the chest, and it is only fair to remember that the man who wears it (with a very thin suit) on the coldest evenings in winter, most probably wears a not very protective soft shirt during the day. As regards spats, cigarettes, and central heating we are with her, and it is really beyond argument that as a people we are growing too fond of being warm. It is not well to be either too hot or too cold, but, broadly speaking, it is better for health in the end to be too cold than too hot. We all have our favourite vices, and the man who would scorn to wear a muffler will thoroughly enjoy his early cup of tea and not think himself a sybarite. Dr. Jane Walker twists the knife in the wound by comparing men's softness with women's hardness. She "tremendously" admires the modern girl who wears "the thinnest of thin silk stockings." No man who is not a pretentious prude will deny the allurement of silk stockings—they even disturbed Dr. Johnson—but is it quite certain that they mean hardihood, and that fashion has nothing to do with their prevalence? We can imagine that there are men cynical enough to suggest that women would forswear stockings altogether if fashion decreed that they were no longer to be worn. But we need have no fear on the subject. It is the business of fashion to popularise new cothes and not to discourage them altogether.

### THE COST OF STREET COLLECTIONS.

HOSPITALS are so closely concerned in the collection of money, especially when it is gathered in the streets, that the annual reports of the London Mendicity Society on street collections must needs be scanned by their governing bodies with some amount of concern. The sixth of these reports, which has just been issued, shows that the average cost of street collections in 1923 was 16 per cent.—3¼ per cent. higher than in the preceding year—on a total of £186,966. This is a tragic fall from 1919 when the total was £416,000, with expenses amounting only to 12 per cent. The country was then in the midst of an illusory "boom," before heavy taxation, restricted trade, and vast unemployment had begun to be felt as they are felt now. Last year the Combined Hospital Collection cost 24½ per cent.—five shillings in the pound—while the National Maternity Collection cost 55, and the Ypres League 90 per cent.! There were actually 226 collections last year which realised less than £100—a circumstance which plainly suggests that much stricter regulation is urgently needed. The report points out that there is "a tendency to form small local collecting societies, especially for hospitals, in poorer districts" in which it is easy to incur an undue amount of expense in organising processions and hiring bands, and that moving demonstrations and perambulating collectors are an annoyance to the public, and consequently unpopular. Hospitals will, therefore, be wise to keep a tight rein over helpers whose zeal and enthusiasm are not only not according to discretion, but are far removed from wisdom.

### DANGERS OF ARTIFICIAL MALARIA.

THE teaching, which now amounts almost to a cult, that certain diseases of the central nervous system can be appreciably alleviated, if not actually cured, by giving the patient malaria, is a matter which the rest of the community cannot afford to ignore. The patient may perhaps get from the fire into the frying-pan by exchanging a greater for a less disease; but if in the process he passes malaria on to his neighbours, their feelings will assuredly be as hostile as those of the owners of the Gadarene swine. This matter is already regarded as so serious in Denmark that the medical authorities in that country have recently had to issue a circular to the medical profession pointing out the precautions that must be taken to prevent mosquitoes from stinging the subjects of artificial malaria and then passing the infection on to other persons. The Danish authorities have decreed that this malarial treatment must not be undertaken by any physician without a special licence, and only in exceptional cases may it be carried out save in a hospital licensed to inoculate patients with malaria. In these hospitals candidates for malarial inoculation must be confined to a mosquito-proof ward in the upper storeys of the building, and the patients must not walk abroad at those hours of the day when mosquitoes usually feed. It has further been decided that they must not be discharged from hospital before they have undergone a systematic course of quinine, and examinations of the blood, repeated twice a week, have failed for three weeks to show the parasite of malaria. As a further precaution against the spread of malaria, these patients have to be notified to the local medical authorities after discharge from hospital, full particulars being given of the patient's whereabouts. This is a matter to which our own medical authorities might well pay some attention.

### "DRAGONS' TEETH" AS CURES.

CHINESE prescriptions may not begin with the words "Fee, fi, fo, fum," but they appear very often to continue in the familiar strain, save that, instead of making bread of an Englishman's bones, the Celestial physician makes medicine of "dragons' bones" and teeth. His ingenuities in this direction have had attention pointedly called to them by the return to America of Mr. Walter Granger, who conducted the recent Asiatic Expedition, in the course of which he discovered a "mine" of these bones. Fossils of this description form a regular article of the Chinese pharmacopœia. They are administered in various ways—ground up and eaten raw, ground and fried in oil, mixed with wine and so on. They are believed to be specific for nervous diseases and disorders of the heart and liver. Actually, they consist mainly of bicarbonate and acid phosphates of lime, so that what they can really do must be left to physicians to decide. The skulls and bones brought back by Mr. Granger are said by many authorities to represent a fauna inhabiting the forests and foot-hills of south China about two million years ago, and having just that composition in which we might expect to find remains of the ancestors of man as they were in the late Tertiary period. Although the possibility of this discovery was well understood, it has, apparently, not eventuated. These "dragons' bones" represent, however, many points in evolution among lower animals, even if their curative powers towards modern man remain a little doubtful.

### THE HEALTH PLAY.

IT is natural enough that the ingenious land which invented the Health Clown should go a step further and elaborate the Health Play. In America these things are not left to private initiative. The Bureau of Education of the Department of the Interior at Washington has lately issued a pamphlet, written by a registered nurse, on "Dramatics for Health Teaching," containing suggestions for, and photographs of, health plays and a useful bibliography of the subject, and Miss Frances Cook, Director of the Health Crusade of the Chicago Tuberculosis Institute, has been explaining in *The Trained Nurse* how it is done. She has a play of her own called "Seven Keys," which is constructed around the idea of a search for Seven Keys of Health which have been hidden by Ignorance, who refuses to open the City Gate to Good Health. With the help of the Doctor and Nurse (messengers of Knowledge) the children of the city find the keys. These keys are so constructed that, when placed in the gate, they form the double-barred red cross. An educational as well as spectacular feature is thus presented when the children, assisted by Hope and Happiness, use these keys to open the way for Health. The play is supposed to take place in "The City of Waybehind," or, as we should say in the English language, the city of "Behind the Times." In this benighted place Ignorance rules; the children associate with Carelessness, Laziness and Neglect only to find suffering and despair through the many evils that surround them; Bad Air, Poor Food, Dirt, Dark Rooms and Germs follow closely upon their heels. They finally come upon the Road to Knowledge, where they learn, through the Doctor and Nurse, that if they perform certain "health chores" the evils will disappear and the power of Ignorance will be destroyed. The "Health Play" is a picturesque idea which it should not be more difficult to present in a variety of attractive ways in England than in America.

### OLD AT SIXTY!

IT is a curious circumstance that, although the average age at death is increasing with some rapidity, young people are more than ever in the habit of speaking of the middle-aged as "old." Not long ago one of those daily illustrated newspapers which appear to be produced by ignorant persons under thirty for the benefit of still more ignorant persons under twenty, described a gentleman of something over fifty as "the aged peer." And now we have Dr. Jane Walker talking of "an old man of sixty!" It is time we began to realise that in these days, nobody in good health is "old" till he is eighty, at least. We see public men continuing all manner of activities until they are nearer eighty than seventy, and there is good reason to believe that very often they reach a good old age because they continue those activities. We are glad to see that Miss Anna Maud Hallam, in a lecture at Sheffield, has been pouring scorn on the absurd convention that old age must needs begin in middle age. "At a certain period," she says, "generally between forty and fifty years of age, people begin to call attention to the fact that they are growing old, but this is really due to a tendency to laziness." Laziness at fifty! We have little doubt that Miss Hallam is right; but if so, what are we to say of the mental laziness of those who unthinkingly believe these absurdities? The idea that healthy, happy, well occupied people begin to go downhill at fifty is, or ought to be, utterly exploded. To think you are tired and old in middle age is to become tired and old, and the best remedy for such unhealthy imaginings is to take a little more exercise and do a great deal more work. Most of us are very far from being fully employed.

---

### EVERYTHING BUT FOOD.

ATTENTION is being called in India to some curious facts concerning rice. Even in India, where it is a staple food, and where an amazing variety of other uses are also found for it, relatively huge nutritive wastage occurs. This may not, it is true, apply to milled rice as such, but this milling is the cause of the loss. Large numbers of the more backward population prefer to buy whole, natural rice, and they would be wise in doing so were it not for the fact that they then throw away the outer coverings. The more advanced people adopt increasingly the highly-milled product, and in their case the nutritive husk is thrown away for them. Milled, polished rice, from the protein and vitamin point of view, is nothing more than the skeleton of the original unhusked rice, and the nutritive loss to the eater is correspondingly enormous. On the other hand, the various by-products of the rice industry are somewhat striking. The husk, etc., although discarded for human use, is utilised for cattle fodder, and the plant itself is valuable for thatching and for feeding stock. Considerable industries are devoted to making starch and alcohol from broken and low-grade rice. The glutinous portions of rice are used in making certain kinds of cement, and rice bran for fattening and the manufacture of various face powders may be included among the indirect uses. A certain amount of paper is made, but the chief use of rice stubble is to be converted into straw and paste-board. Rice husk can be used as a fuel, and actually runs many of the mills. Without this fortunate possibility it would be impossible to market milled rice at anything approaching its present price. Oil extract from the husk is another development. Thus there are many advantages to be set off against the nutritive wastage which the increasing demand for polished rice implies. There is, however, little doubt that investigation is badly needed of methods by which the superficial husk alone might be removed, and so leave for consumption the whole of the natural rice.

---

### THE ELIMINATION OF DIPHTHERIA.

THE last two seasons having been comparatively mild, as far as the prevalence of diphtheria is concerned, opportunity has occurred to think over the progress made towards real suppression of this disease. Since many, perhaps most, of the deaths from diphtheria are preventable, it is axiomatic that somehow or other this prevention must sooner or later be achieved, and no pains must be spared in the process. At present three lines of attack are open—the recognition of people who convey the germ, the isolation of these persons, and the active immunisation of everyone who is susceptible to the disease. The last procedure implies application of the much discussed Schick test, and, where necessary, immunisation by injection. Now the success of this last, coupled with the momentary quietude of the disease itself, has perhaps led us to relax our search for carriers and contacts who are, so to speak, keeping the infection alive ready for its next real outburst. In some quarters there is certainly an apparent slackening of zeal in swabbing the throats, and particularly the nasal passages, of possibly infected persons. It seems therefore justifiable to remind those responsible for such things that a feeling of false security as far as diphtheria is concerned is very dangerous.

---

### DENTAL CLINICS.

MOST of us realise how bad is the average dental condition of the British people, but few appreciate how early, among the masses, the trouble sets in. Mr. George Thomas, the President of the Dental Officers Group of the Society of Medical Officers of Health, has recently reminded us that large numbers of children have decaying molar teeth at the age of two years onward, and that, at four years old, many teeth are past saving, since only a small percentage of British children preserve their temporary molars up to the age of ten years. Also a great many children have lost their first permanent molar teeth at an age when they are most needed for the mastication of food, and it is admitted that the initiation of gastric disorders in children is the preliminary to tuberculous infection. The crying need is for greater practice of conservative dentistry among the poorer classes. The case for the establishment of dental clinics at which professional men give some part of their time to further the true dental interests of the industrial classes is strong indeed.

---

### HEALTH OF CHICAGO CHILDREN.

THE Chicago Parental School is an institution providing sleeping accommodation by night, as well as instruction by day, for children who have passed through the Juvenile Court, and have been found in need of closer care and supervision than the home, if it exists, can give. This school has recently been subjected to a health survey, a movement which is yearly growing in favour in the U.S.A. The survey was conducted by the Chicago Municipal Tuberculosis Sanatorium, which for several years has acted the part of a troublesome hygienic conscience to Chicago. The report of this survey is in some matters a scathing indictment of the supervision of the physical welfare of the children. It was found that there were no facilities for dental treatment for these 300 to 400 children. The physician in charge received a salary of only £17 a month, and visited the school only twice a week. The report of this health survey has general as well as local interest. It is the business of Chicago alone to see that its institutions for waifs and strays are conducted on humane and intelligent principles. But the report raises the suspicion that the evils discovered may not be peculiar to Chicago, and may flourish elsewhere, even under our very noses.

